# Crowdsourcing the creation of image segmentation algorithms for connectomics

**DOI:** 10.3389/fnana.2015.00142

**Published:** 2015-11-05

**Authors:** Ignacio Arganda-Carreras, Srinivas C. Turaga, Daniel R. Berger, Dan Cireşan, Alessandro Giusti, Luca M. Gambardella, Jürgen Schmidhuber, Dmitry Laptev, Sarvesh Dwivedi, Joachim M. Buhmann, Ting Liu, Mojtaba Seyedhosseini, Tolga Tasdizen, Lee Kamentsky, Radim Burget, Vaclav Uher, Xiao Tan, Changming Sun, Tuan D. Pham, Erhan Bas, Mustafa G. Uzunbas, Albert Cardona, Johannes Schindelin, H. Sebastian Seung

**Affiliations:** ^1^UMR1318 French National Institute for Agricultural Research-AgroParisTech, French National Institute for Agricultural Research Centre de Versailles-Grignon, Institut Jean-Pierre BourginVersailles, France; ^2^Howard Hughes Medical Institute, Janelia Research CampusAshburn, VA, USA; ^3^Center for Brain Science, Harvard UniversityCambridge, MA, USA; ^4^Swiss AI Lab IDSIA (Dalle Molle Institute for Artificial Intelligence) Universitá Della Svizzera Italiana, Scuola Universitaria Professionale Della Svizzera ItalianaLugano, Switzerland; ^5^Department of Computer Science, ETH ZurichZurich, Switzerland; ^6^Scientific Computing and Imaging Institute, University of UtahSalt Lake City, UT, USA; ^7^Imaging Platform, Broad InstituteCambridge, MA, USA; ^8^Department of Telecommunications, Faculty of Electrical Engineering and Communication, Brno University of TechnologyBrno, Czech Republic; ^9^School of Engineering and Information Technology, University of New South WalesCanberra, ACT, Australia; ^10^Digital Productivity Flagship, Commonwealth Scientific and Industrial Research OrganisationNorth Ryde, NSW, Australia; ^11^Department of Biomedical Engineering, The Institute of Technology, Linkoping UniversityLinkoping, Sweden; ^12^Computer Science Department, Rutgers UniversityNew Brunswick, NJ, USA; ^13^Laboratory for Optical and Computational Instrumentation, University of Wisconsin-MadisonMadison, WI, USA; ^14^Princeton Neuroscience Institute and Computer Science Department, Princeton UniversityPrinceton, NJ, USA

**Keywords:** connectomics, electron microscopy, image segmentation, machine learning, reconstruction

## Abstract

To stimulate progress in automating the reconstruction of neural circuits, we organized the first international challenge on 2D segmentation of electron microscopic (EM) images of the brain. Participants submitted boundary maps predicted for a test set of images, and were scored based on their agreement with a consensus of human expert annotations. The winning team had no prior experience with EM images, and employed a convolutional network. This “deep learning” approach has since become accepted as a standard for segmentation of EM images. The challenge has continued to accept submissions, and the best so far has resulted from cooperation between two teams. The challenge has probably saturated, as algorithms cannot progress beyond limits set by ambiguities inherent in 2D scoring and the size of the test dataset. Retrospective evaluation of the challenge scoring system reveals that it was not sufficiently robust to variations in the widths of neurite borders. We propose a solution to this problem, which should be useful for a future 3D segmentation challenge.

## 1. Introduction

Electron microscopy (EM) has revealed novel facts about synapses and other subcellular structures in the mammalian nervous system (Bourne and Harris, 2012). Serial EM has been most famously used to reconstruct the connectivity of the *Caenorhabditis elegans* nervous system (White et al., 1986; Jarrell et al., 2012). More recent improvements in this technique have led to imaging of much larger volumes of brain tissue, and exciting insights into invertebrate nervous systems (Bumbarger et al., 2013; Takemura et al., 2013; Kasthuri et al., 2015), and mammalian neural circuits (Briggman et al., 2011; Tapia et al., 2012; Helmstaedter et al., 2013; Kim et al., 2014). However, these recent studies also point to an important need for the development of new computational technology to aid the analysis of EM imagery of brain tissue.

In a recent study, about 1000 neurons were reconstructed from a mouse retina using 20,000 h of human labor (Helmstaedter et al., 2013). In spite of this great effort, the reconstructed retinal volume was just 0.1 mm on each side, only large enough to encompass the smallest types of retinal neurons. This study employed semiautomated methods, using advances in machine learning to automate most of the reconstruction (Jain et al., 2010b). Without the automation, the reconstruction would have required 10–100× more human effort. To reconstruct larger volumes, it is critical to improve the accuracy of computer algorithms and thereby reduce the amount of human labor required by semiautomated systems. Ideally, the need for human interaction will be progressively eliminated, gradually enabling fully automated tracing with eventual proof-reading of its results.

To accelerate research in machine learning, we adopted a crowdsourcing approach. Previously, research on serial EM image analysis was mainly confined to a few researchers who were direct collaborators with neuroscientists who acquired the images. We sought to attract talent from the “crowd” through the first serial EM image segmentation challenge. Netflix has used a crowdsourcing approach to improve the accuracy of automated movie recommendations^1^, and the Heritage Provider Network to improve prediction of unnecessary hospitalizations using patient data^2^. Kaggle and other online marketplaces for such machine learning competitions have been established. Crowdsourcing has also been employed to drive innovation in scientific problems, such as biological sequence analysis (Lakhani et al., 2013) and particle tracking in microscopy images (Chenouard et al., 2014).

Both competitive and cooperative mechanisms are used in crowdsourcing (Bullinger et al., 2010). In the first phase of our challenge, competitive mechanisms were dominant. This phase lasted for 4 months and ended with a workshop at the International Symposium on Biomedical Imaging 2012 conference (ISBI'12). The winning entry, a deep convolutional network, attained over 2.5× improvement in accuracy relative to the start of the challenge. Notably, the winning entry came from a team (IDSIA) having no prior experience with EM images, demonstrating our success in recruiting new talent from the crowd.

Seven teams publicly divulged their algorithms at the workshop, and this cooperative interaction commenced a second phase of the challenge. IDSIA released the results of processing the EM images by their winning entry, and another team (SCI) built on these results to attain further improvement of over 2.5×. This demonstrated the power of cooperative mechanisms in crowdsourcing algorithm design.

A good scoring system is important for successful crowdsourcing. Rand and information theoretic scores have been proposed for segmentation of EM images (Turaga et al., 2009; Nunez-Iglesias et al., 2013). This paper compares the two scoring systems using empirical data obtained from the challenge, and exposes some of their strengths and weaknesses.

Since the ISBI'12 workshop, convolutional networks have become accepted as a standard computational tool for EM image segmentation. This is analogous to a similar acceptance of deep convolutional networks (also known as “deep learning”) as the leading approach to visual object recognition, which was triggered by the ImageNet challenge (Krizhevsky et al., 2013). In the fall of 2012, an algorithm based on a deep convolutional neural network won the competition by a significant margin, dropping the existing error rate from 25.8% (in 2011) to only 16.4%. This result made a real impact in the field of image and object recognition and is considered today a turning point in machine vision (Russakovsky et al., 2014).

To summarize, our contributions in this paper are:

- The first public competition in the field of image segmentation for brain connectomics. This competition had the dual goal of attracting new researchers to the field of connectomics, and improving the state-of-the-art for EM neuron segmentation.
- A crowdsourcing structure, combining competition and cooperation through a website and forum, promoting novel algorithmic solutions from the participants.
- Novel analysis and comparison of segmentation evaluation metrics, both from theoretical and empirical perspectives.
- A novel evaluation metric that overcomes problems in earlier metrics which can be used in future 2D and 3D segmentation challenges.

## 2. Materials and methods

### 2.1. Image acquisition

The training data is a set of 30 consecutive images (512 × 512 pixels) from a serial section Transmission Electron Microscopy (ssTEM) dataset of the *Drosophila* first instar larva ventral nerve cord (VNC; Cardona et al., 2010). The imaged volume measures 2 × 2 × 1.5 μ, with a resolution of 4 × 4 × 50 nm/pixel. The images were captured using Leginon (Suloway et al., 2005) to drive a FEI electron microscope equipped with a Tietz camera and a goniometer-powered mobile grid stage, with a magnification of 5600× binned at 2, which delivers the 4 × 4 nm per pixel resolution. This imaging technique delivers image volumes in a highly anisotropic manner, i.e., the x- and y-directions have a high resolution, whereas the z-direction has a low resolution limited by physical sectioning of the tissue block. Electron microscopy produces the images as a projection of the whole section, so some of the membranes that are not orthogonal to the cutting plane can appear blurred.

### 2.2. Training and test data sets

**Figure 1:**
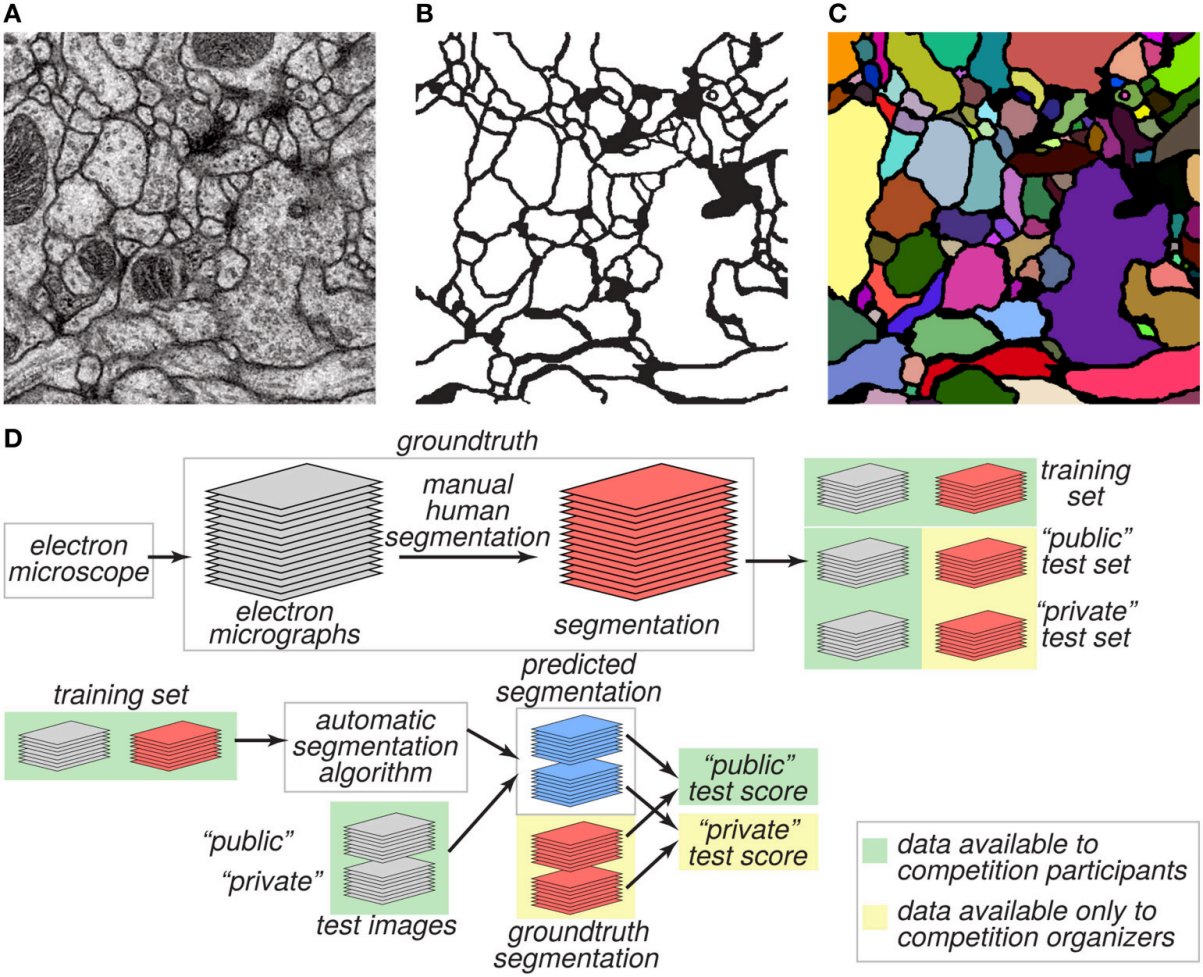
**Challenge datasets**. **(A)** EM image of the ventral nerve cord of a larval *Drosophila*. **(B)** Boundary map annotated by human experts. **(C)** Segmentation into neurite cross-sections. **(D)** The annotated dataset was split into training and test sets and distributed publicly. Ground truth labels for the test set were withheld and used to evaluate the predictive performance of candidate algorithms.

The goal of the challenge was to find algorithms for transforming a grayscale EM image (Figure 1A) into an accurate boundary map (Figure 1B), defined as a binary image in which “1” indicates a pixel inside a cell, and “0” indicates a pixel at a boundary between neurite cross sections. A boundary map is equivalent to a segmentation of the image (Figure 1C).

Boundary detection is challenging because many boundaries look fuzzy and ambiguous. Furthermore, only boundaries between neurites should be detected, and those of intracellular organelles like mitochondria and synaptic vesicles should be ignored.

We created two datasets, training and test, for evaluating performance on this task (Figure 1D). These two datasets were 30 grayscale images each, like the one of Figure 1A. The *ground truth* boundary maps for the training images were created by one coauthor (AC) who manually segmented each neurite of the training volume by manually marking its borders on each 2D plane. Although we refer to the human expert annotation as *ground truth* for simplicity as is common in machine learning, it is important to note that the human annotation may itself contain errors relative to the true underlying biological reality. The ground truth boundary maps for the test images were created by two other coauthors (IA and DB), who independently segmented the whole test volume. AC and IA manually delineated the neurite boundaries using the open-source software TrakEM2 (Cardona et al., 2012), while DB used the freely-available software VAST^3^. The final test labels were created as a consensus of the two test boundary maps. With that purpose, the labels from IA (H1) were visually inspected and compared with the labels of DB (H2). Whenever a disagreement (usually an object split or merger) was found, a manual correction was performed to guarantee the 3D object continuity.

The training dataset was made publicly available, so that participants in the challenge could use it for developing algorithms. From the test dataset, only the grayscale images were made publicly available. The ground truth boundary maps of the test images were kept private and only a secret portion of them were used to calculate the public test score (Figure 1D). The participants submitted predicted boundary maps for the test images. The organizers scored the predicted boundary maps by comparing them to the withheld ground truth.

### 2.3. Measures of segmentation accuracy

Scoring boundary maps may sound straightforward, but is non-trivial. Ideally, the score of an algorithm should indicate its potential utility in practical applications. In connectomics, a boundary detection algorithm is generally embedded in a semiautomated system that enables human experts to segment images by correcting mistakes of the algorithm (Chklovskii et al., 2010; Kim et al., 2014). Therefore, each algorithm could be scored by a “nuisance metric,” defined as the amount of human effort required for correction. However, the nuisance metric is cumbersome since it cannot be computed automatically, and it depends on the details of the semiautomated system used and on the humans involved. Therefore, we sought some approximation to the nuisance metric that can be computed more easily.

**Figure 2:**
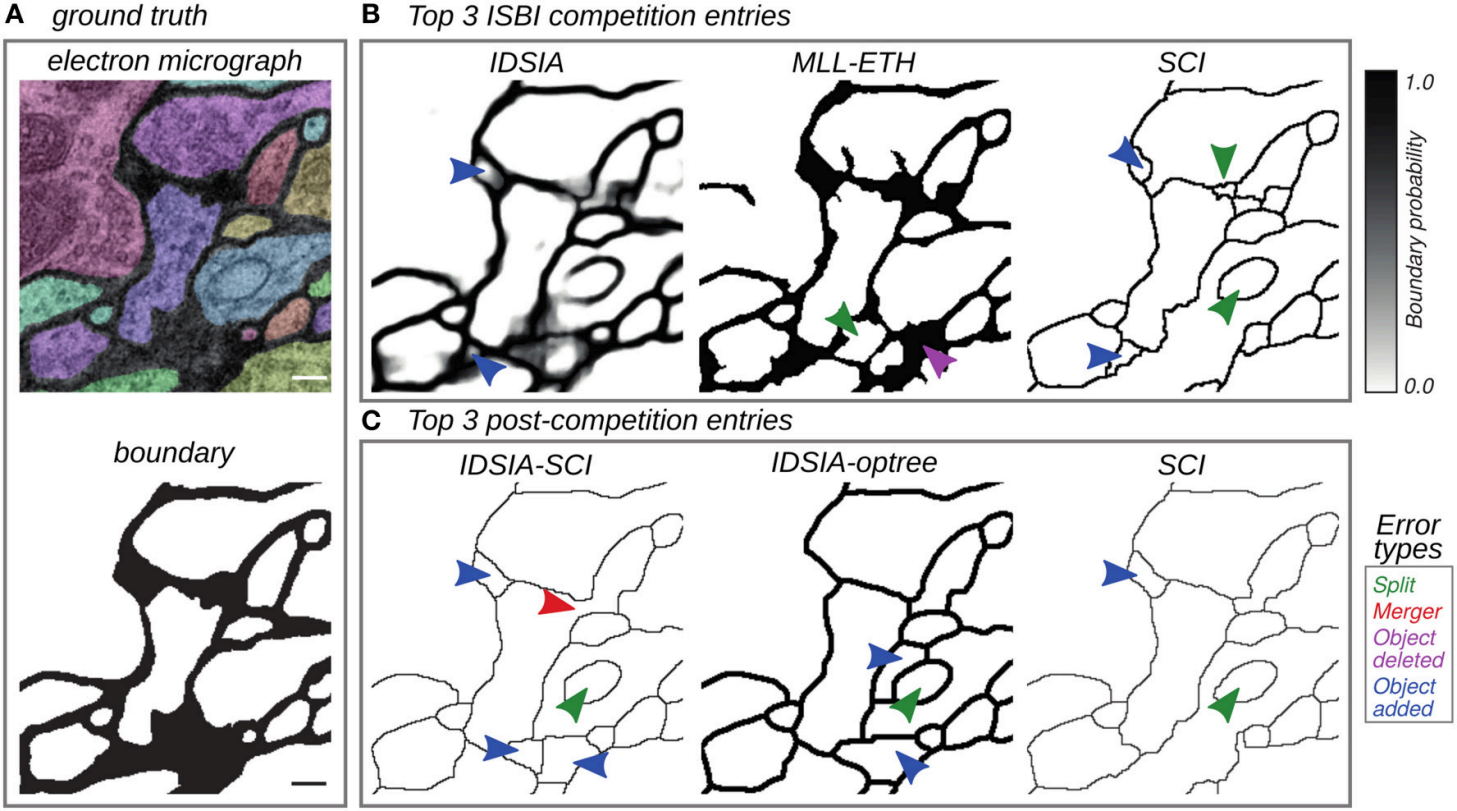
**Top entries from the competition (Section 3.2) and cooperation (Section 3.3) phases of the challenge**. **(A)** Electron micrograph with overlaid segmentation and corresponding boundary map. **(B)** Boundary maps of the top 3 submissions at the time of ISBI'12. **(C)** Boundary maps of the top 3 submissions in the cooperation phase. Segmentation errors are marked by arrows colored based on the type of mistake: split (green), merge (red), omission (magenta), and addition (blue). Scale bar = 100 nm.

Human effort is required to correct split errors, in which one neuron is incorrectly split into two segments, and merge errors, in which two neurons are incorrectly merged into one segment (Figure 2). Therefore, quantifying split and merge errors should provide some approximation to the nuisance metric.

Given a binary boundary labeling of an image, the easiest measure of segmentation performance to compute is a local pixel-wise boundary prediction error (*pixel error)*. Unfortunately, pixel error considers only whether or not a given pixel was correctly classified as a boundary pixel, without concern to the ultimate effect of that prediction on the resulting image segmentation. For example, expanding, shrinking or translating a boundary between two neurons would not cause splits or mergers, but incur a large pixel error. Further, while a gap of even a single pixel in the boundary between two neurons would cause a merge error, it might only incur a very small pixel error as a fraction of the total number of pixels in the image. The first of these problem has been mitigated by the Berkeley metrics (Martin et al., 2004), however the second problem still remains, ultimately leaving the pixel error family of metrics inadequate.

Several candidate non-local, region-based metrics have been suggested to solve the problems associate with the naive pixel error. *Rand error* has been proposed as a metric of segmentation performance (Unnikrishnan et al., 2007; Arbelaez et al., 2011), and has also been used as an objective function for directly optimizing the performance of machine learning algorithms (Turaga et al., 2009). *Variation of information* (Meilǎ, 2005) is closely related to Rand error (see Section S3), and has also been used as a segmentation metric (Arbelaez et al., 2011), and as an objective function (Kroeger et al., 2013). *Warping error*, based on digital topology, has been proposed as a metric and used as a cost function for machine learning (Jain et al., 2010a).

After evaluating all of these metrics and associated variants (see Supplementary Material), we found empirically that specially normalized versions of the Rand error *V*^Rand^ (Equation 3), and Variation of Information *V*^Info^ (Equation 6) best matched our qualitative judgements of segmentation quality. We show empirically that of these two popular metrics, *V*^Rand^ is more robust than *V*^Info^, and for a theoretical analysis comparing these two evaluation metrics, please see Section S3.

### 2.4. Foreground-restricted rand scoring *V*^Rand^

Any boundary map can be transformed into a segmentation by finding connected components. Suppose that *S* is the predicted segmentation and *T* is the ground truth segmentation. Define *p*_*ij*_ as the probability that a randomly chosen pixel belongs to segment *i* in *S* and segment *j* in *T*. This joint probability distribution satisfies the normalization condition $\underset{ij}{\sum }p_{ij}=1$. The marginal distribution $s_{i}=\underset{j}{\sum }p_{ij}$ is the probability that a randomly chosen pixel belongs to segment *i* in *S*, and the marginal distribution $t_{j}=\underset{i}{\sum }p_{ij}$ is defined similarly.

Two randomly chosen pixels belong to the same segment in *S* and the same segment in *T* with probability $\underset{ij}{\sum }p_{ij}^{2}$. This quantity is expected to be larger when *S* and *T* are more similar. We will use it to define measures of similarity between *S* and *T*, using appropriate normalizations to constrain these measures to the range [0, 1]. For example,

(1) $$V_{\text{split}}^{\text{Rand}}=\frac{\sum_{ij}p_{ij}^{2}}{\sum_{k}t_{k}^{2}}$$

is the probability that two randomly chosen voxels belong to the same segment in *S*, given that they belong to the same segment in *T*. We will call this the Rand split score, because it is higher when there are fewer split errors. We also define the Rand merge score

(2) $$V_{\text{merge}}^{\text{Rand}}=\frac{\sum_{ij}p_{ij}^{2}}{\sum_{k}s_{k}^{2}}$$

as the probability that two randomly chosen voxels belong to the same segment in *T*, given that they belong to the same segment in *S*. The merge score is higher when there are fewer merge errors.

For a single score that includes both split and merge errors, we can use the weighted harmonic mean

(3) $$V_{\alpha }^{\text{Rand}}=\frac{\sum_{ij}p_{ij}^{2}}{\alpha \sum_{k}s_{k}^{2}+(1-\alpha )\sum_{k}t_{k}^{2}}$$

We will define the Rand F-score as α = 0.5, which weights split and merge errors equally. The values α = 0 and α = 1 correspond to the individual split and merge scores above. More generally, one could choose α depending on which kind of error is more time-consuming for humans to correct, or is more detrimental to the scientific investigation.

The split and merge scores can be interpreted as precision and recall in the classification of pixel pairs as belonging to the same segment (positive class) or different segments (negative class). We use the term “Rand” because the Rand F-score is closely related to the Rand index, which was previously used to quantify performance at clustering (Rand, 1971) and image segmentation (Unnikrishnan et al., 2007; Arbelaez et al., 2011). The Rand index was also used as an objective function for machine learning of image segmentation (Turaga et al., 2009).

To compute the above scores, each boundary map was transformed into a segmentation by regarding connected components of “1”s as segments. In addition, we followed the convention that every “0” pixel was regarded as a segment containing just one pixel.

One complication for scoring is that algorithms (and humans) often differ in the widths they ascribe to the borders between cells. Such minor differences are unimportant, and an ideal scoring system should be robust to them. Therefore, we excluded border pixels in the ground truth boundary map from the computation of Rand scores. The foreground-restricted scores were empirically found to be less sensitive to small border variations. We chose not to exclude border pixels in the predicted boundary map, because this modification might have made the score susceptible to exploitation by participants.

The organizers chose the foreground-restricted Rand F-score as the official ranking system of the competition. Code for computing this score was made available to the participants. Using this code, participants could readily score their algorithms on the training set. Participants could not easily score their algorithms on the test set, as the ground truth boundary maps for the test set were kept private by the organizers. To help preserve impartiality of evaluation, the organizers (IA, ST, JS, AC, and HS) did not participate in the challenge.

### 2.5. Information theoretic scoring *V*^Info^

After receiving many submissions, we decided to retrospectively evaluate our scoring system by empirical means. Information theoretic scoring has been proposed as an alternative to Rand scoring (Nunez-Iglesias et al., 2013). We decided to compare the two scoring systems on all submissions.

The mutual information $I\left(S;T\right)=\underset{ij}{\sum }p_{ij}\log p_{ij}-\underset{i}{\sum }s_{i}\log s_{i}-\underset{j}{\sum }t_{j}\log t_{j}$ is a measure of similarity between *S* and *T*. This can be used to define related measures of similarity that are normalized to the range between 0 and 1. Dividing by the entropy $H\left(S\right)=-\underset{i}{\sum }s_{i}\log s_{i}$ yields the information theoretic split score

(4) $$V_{\text{split}}^{\text{info}}=\frac{I(S;T)}{H(S)}$$

the fraction of information in *S* provided by *T*. Dividing by *H*(*T*) yields the information theoretic merge score

(5) $$V_{\text{merge}}^{\text{info}}=\frac{I(S;T)}{H(T)}$$

the fraction of information in *T* provided by *S*. Both scores are non-negative and upper bounded by unity, due to well-known properties of mutual information. The weighted harmonic mean of the two scores is

(6) $$V_{\alpha }^{\text{info}}\;=\;\frac{I(S;T)}{\left(1-\alpha \right)H(S)+\alpha H(T)}$$

We will refer to α = 0.5 as the information theoretic F-score. The values α = 0 and α = 1 correspond to the individual split and merge scores above. Other choices of α may be used if split and merge errors have differing importance.

The information theoretic F-score is closely related to the variation of information, which has been proposed as a metric for clustering (Meilǎ, 2005) and image segmentation (Arbelaez et al., 2011), and as an objective function for machine learning of segmentation (Kroeger et al., 2013). For the sake of comparison, the foreground-restricted information theoretic score is shown in all the results presented here.

## 3. Results

### 3.1. Rankings at the time of ISBI'12

The first column of Table 1 gives the Rand scores of all 13 teams who entered before ISBI'12. Teams submitted multiple entries over time, so the best submission from each team before ISBI'12 is shown. Based on this ranking, IDSIA was declared the winner at the ISBI'12 workshop.

**Table 1:** **Best Rand and information theoretic scores of all teams and the human experts using the undisclosed test set at ISBI**.

**Method**	***V*^Rand^**	***V*^Info^**
Human 1 vs. consensus	0.997 ± 0.001	0.997 ± 0.001
human 2 vs. consensus	0.971 ± 0.003	0.941 ± 0.002
IDSIA	0.944 ± 0.011	0.968 ± 0.002
BlackEagles	0.929 ± 0.008	0.916 ± 0.003
MLL-ETH	0.927 ± 0.008	0.923 ± 0.004
SCI	0.915 ± 0.016	0.967 ± 0.003
CellProfiler	0.904 ± 0.015	0.937 ± 0.006
Harvard	0.892 ± 0.017	0.947 ± 0.004
CoMPLEX	0.877 ± 0.019	0.903 ± 0.008
UCL	0.860 ± 0.020	0.939 ± 0.005
TSC+PP	0.843 ± 0.012	0.838 ± 0.006
IMMI	0.826 ± 0.022	0.862 ± 0.008
CLP	0.809 ± 0.018	0.846 ± 0.005
Freiburg	0.800 ± 0.026	0.825 ± 0.005
NIST	0.730 ± 0.021	0.757 ± 0.007

*Mean and standard error are computed over 20 test images not used for the public leaderboard*.

**Figure 3:**
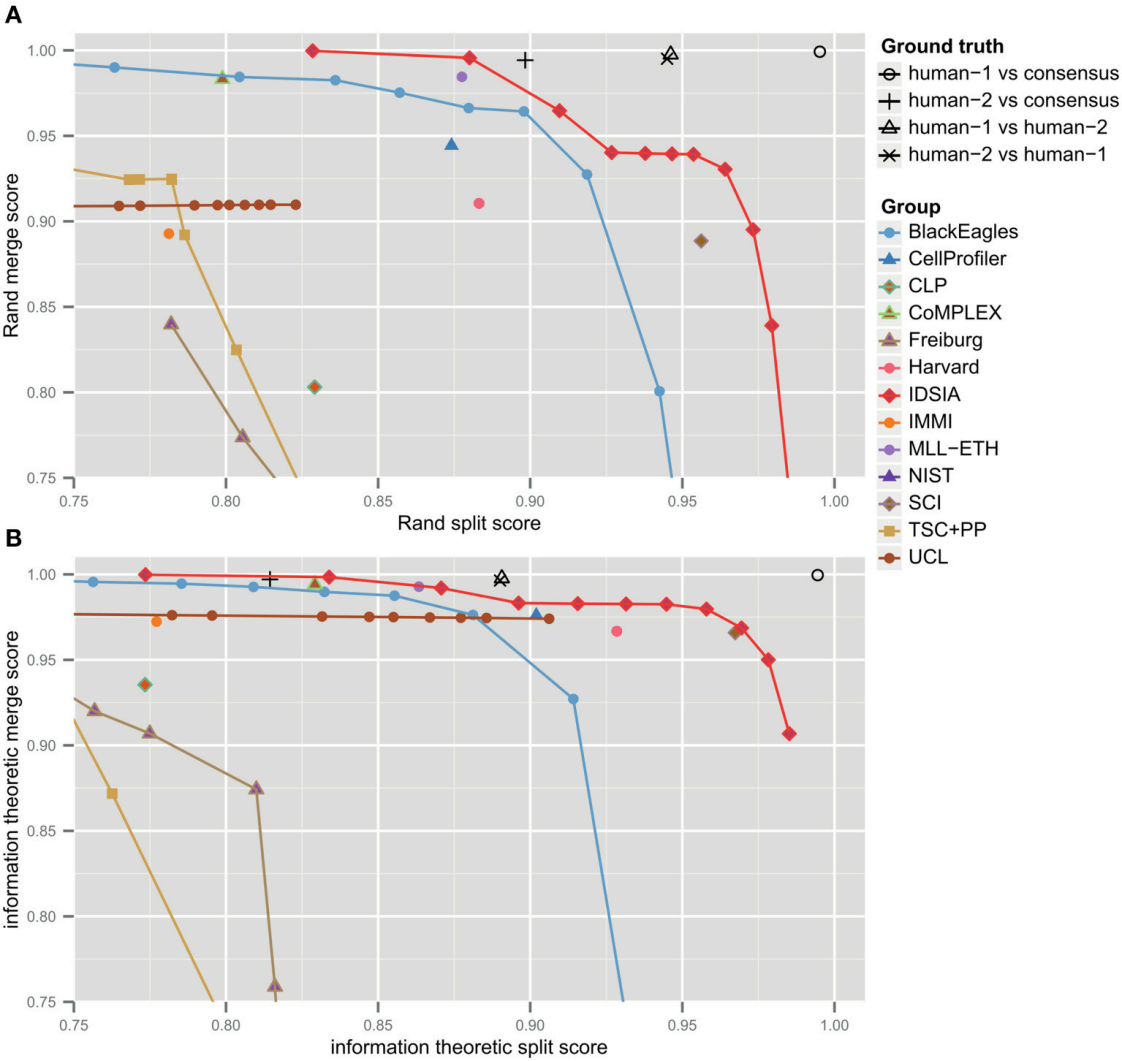
**Merge vs. split scores for submissions prior to competition deadline**. Upper right hand corner corresponds to perfect performance. **(A)** Rand scores of Equations (1, 2), **(B)** information theoretic scores of Equations (4, 5).

The F-score is an aggregate of split and merge scores (Equation 3). These are plotted in Figure 3A to provide more information about algorithm performance (Figure 3B shows the same values based on the information theoretic score). The upper right hand corner corresponds to perfect performance. For the teams that submitted probabilistic boundary maps, performance is represented by a curve, each point of which corresponds to one value of the threshold used to obtain a deterministic (binary-valued) boundary map. For each of these teams, the values in Table 1 are given for the location on the curve that achieved maximal F-score.

For each non-IDSIA submission, there exists some point on the IDSIA curve with superior split and merge scores. In this sense, IDSIA dominated all other teams in Table 1. However, there exists no single point on the IDSIA curve that is strictly better than all other algorithms.

The IDSIA entry was a deep convolutional network (Ciresan et al., 2012). This approach used “end-to-end learning,” meaning that the raw image was fed directly to a complex pattern classifier. Other teams also used machine learning approaches, but some relied heavily on hand-designed features, which were used as inputs to a simple pattern classifier. In total, seven teams provided information about their algorithms to the organizers and presented their work at the workshop. A brief description of each method can be found in the Supplementary Material.

### 3.2. Competition yielded over 2.5× improvement

**Figure 4:**
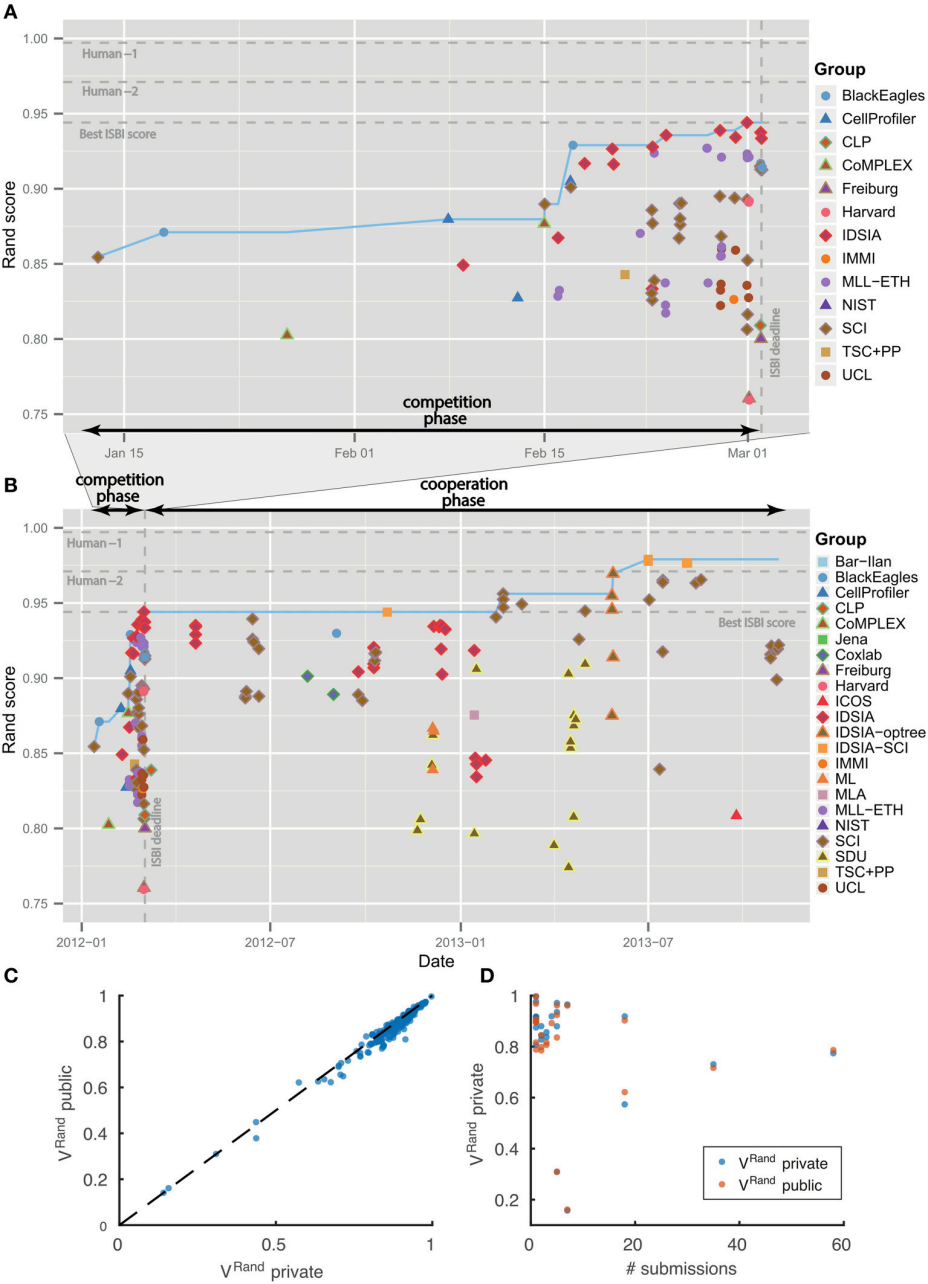
**Evolution of Rand score over time. No overfitting**. **(A)** Competition phase prior to ISBI'12 workshop. **(B)** Cooperation phase. Individual submissions are colored by team. The dotted blue line shows the best Rand score achieved by that date. **(C,D)** Score differences between private and public test datasets.

Table 1 summarizes the results of the first four months of the challenge, which we will call the “competition phase.” The challenge was announced starting on October 25, 2011 through publicity surrounding the ISBI'12 conference, email to the Fiji-ImageJ and ImageWorld lists, and the MICCAI Grand Challenges in Biomedical Image Analysis^4^. Teams immediately began registering for the challenge and downloading the datasets. On January 11, 2012, no submission had yet been received, so the competition deadline was postponed from February 1 to March 1. The first submission was received from SCI on January 13, 2012. The organizers posted scores of all submissions on a leaderboard that was publicly accessible from the challenge website. Over the course of the competition phase, six different teams held first place in the ranking. IDSIA took first place on February 24, 2012, and held this position until the competition deadline on March 1, 2012 (see Figures 4A,B).

The leaderboard scores were computed using 10 images drawn from the 30 images in the test set. Since participants received multiple scores over the course of the competition, there was effectively some opportunity to train on the test set. To see whether overtraining had occurred, the scores in Table 1 were computed using the remaining 20 images from the test set. Figures 4C,D shows that the scores on the 10 and 20 images are indeed very similar.

Before the ISBI'12 workshop, 32 out of 86 submissions were from 8 out of 13 teams with no prior publications in the area of segmenting EM images. Seven out of the top 10 submissions, including the winning submission (IDSIA), came from these inexperienced teams.

The winning submission (0.944 ± 0.012) scored 2.6× closer to perfect (1.0) than the first submission (0.854 ± 0.022), showing that substantial progress was achieved during the competition phase. The difference is statistically significant (Wilcoxon signed rank test, *p* < 0.0001). The winning submission was 2.2× closer to perfect than the median score (0.877 ± 0.019) over all submissions in the competition phase. This is also a statistically significant difference (Wilcoxon signed rank test, *p* < 0.0036).

### 3.3. Post-deadline cooperation yielded over 2.5× improvement

All 13 competition participants were invited to speak at the ISBI'12 workshop. Seven teams agreed to submit papers and gave presentations about their methods. This kicked off a new “cooperation phase” of the challenge in which many participants publicly shared their results and software implementation. In particular, IDSIA publicly released the boundary maps of their winning entry.

Much of the cooperation happened through an online discussion forum^5^ created for the challenge. There were 87 postings as of November 4, 2013. In the competition phase, postings were mainly questions to the organizers. In the cooperation phase, participants used the forum to share their opinions, but also their results and some times even the code they used during the competition.

As of November 4, 2013, there were 185 submissions and 22 teams listed on the leaderboard (Table 2). Apart from the nine new teams, four teams from the competition phase remained active: IDSIA, SCI, BlackEagles and CLP. Two new teams were combinations of individual teams and since they used the probability maps made public by IDSIA, it was agreed to include “IDSIA” in their official group names. The top submission for instance (IDSIA-SCI) was a combination of IDSIA boundary maps with SCI post-processing. The Rand F-score of IDSIA-SCI was 2.7× closer to perfect than IDSIA alone (Table 2, first column). This improvement was statistically significant (Wilcoxon signed rank test, *p* < 0.0041). Interestingly, this gain was about the same as that achieved by the competition phase. Only IDSIA-optree made a significant improvement as well (Wilcoxon signed rank test, *p* < 0.05).

**Table 2:** **Best Rand and information theoretic scores (before and after border thinning) of all teams and the human experts using the undisclosed test set as of November 4, 2013**.

**Method**	***V*^Rand^**	***V*^Info^**	***V*^Rand^ (thinned)**	***V*^Info^ (thinned)**
Human 1 vs. consensus	0.997 ± 0.001	0.997 ± 0.001	0.998 ± 0.001	0.999 ± 0.001
Human 2 vs. consensus	0.971 ± 0.003	0.941 ± 0.002	0.990 ± 0.002	0.989 ± 0.001
IDSIA-SCI	0.979 ± 0.005	0.988 ± 0.002	0.979 ± 0.005	0.988 ± 0.002
IDSIA-optree	0.969 ± 0.006	0.977 ± 0.003	0.972 ± 0.006	0.984 ± 0.002
SCI	0.966 ± 0.006	0.984 ± 0.002	0.968 ± 0.006	0.984 ± 0.002
IDSIA	0.944 ± 0.011	0.969 ± 0.002	0.978 ± 0.004	0.988 ± 0.001
BlackEagles	0.930 ± 0.009	0.941 ± 0.003	0.973 ± 0.005	0.983 ± 0.002
MLL-ETH	0.927 ± 0.008	0.926 ± 0.003	0.968 ± 0.006	0.981 ± 0.002
SDU	0.909 ± 0.011	0.926 ± 0.004	0.942 ± 0.008	0.974 ± 0.003
CellProfiler	0.904 ± 0.015	0.937 ± 0.006	0.915 ± 0.015	0.958 ± 0.005
Coxlab	0.901 ± 0.012	0.936 ± 0.006	0.939 ± 0.012	0.976 ± 0.003
Harvard	0.892 ± 0.017	0.944 ± 0.006	0.907 ± 0.016	0.957 ± 0.003
CoMPLEX	0.877 ± 0.019	0.903 ± 0.008	0.890 ± 0.018	0.947 ± 0.005
MLA	0.875 ± 0.016	0.885 ± 0.004	0.916 ± 0.016	0.964 ± 0.004
ML	0.867 ± 0.016	0.879 ± 0.006	0.911 ± 0.016	0.958 ± 0.003
UCL	0.860 ± 0.020	0.939 ± 0.005	0.863 ± 0.020	0.948 ± 0.005
TSC+PP	0.843 ± 0.012	0.839 ± 0.006	0.922 ± 0.013	0.961 ± 0.005
CLP	0.839 ± 0.024	0.885 ± 0.008	0.869 ± 0.024	0.940 ± 0.006
IMMI	0.826 ± 0.022	0.862 ± 0.008	0.876 ± 0.020	0.948 ± 0.005
ICOS	0.809 ± 0.018	0.838 ± 0.011	0.883 ± 0.015	0.936 ± 0.004
Freiburg	0.800 ± 0.026	0.839 ± 0.007	0.835 ± 0.027	0.928 ± 0.006
NIST	0.730 ± 0.021	0.757 ± 0.007	0.796 ± 0.020	0.851 ± 0.006
Computer Vision Jena	0.709 ± 0.024	0.768 ± 0.012	0.832 ± 0.022	0.904 ± 0.007
Bar-Ilan	0.701 ± 0.034	0.792 ± 0.011	0.773 ± 0.032	0.872 ± 0.012

*For each team, the submission with the highest score is chosen for each column. The values were computed as the mean and standard error over the n = 20 test images that were not used in the public leaderboard*.

### 3.4. Robustness of scoring to border variations

On theoretical grounds, both Rand and information theoretic scoring are closely related to the nuisance metric, and to each other. Therefore, we expected similar rankings to emerge from the comparison, but this turned out not to be the case. According to Table 1, IDSIA would still have been declared the winner at the ISBI'12 workshop by information theoretic scoring. However, SCI would have moved up to second place and the difference between IDSIA and SCI is not statistically significant. BlackEagles and MLL-ETH would have dropped from 2nd and 3rd place in the Rand rankings to 7th and 6th in the information theoretic rankings. Such differences cast doubt on the quality of both scoring systems.

**Figure 5:**
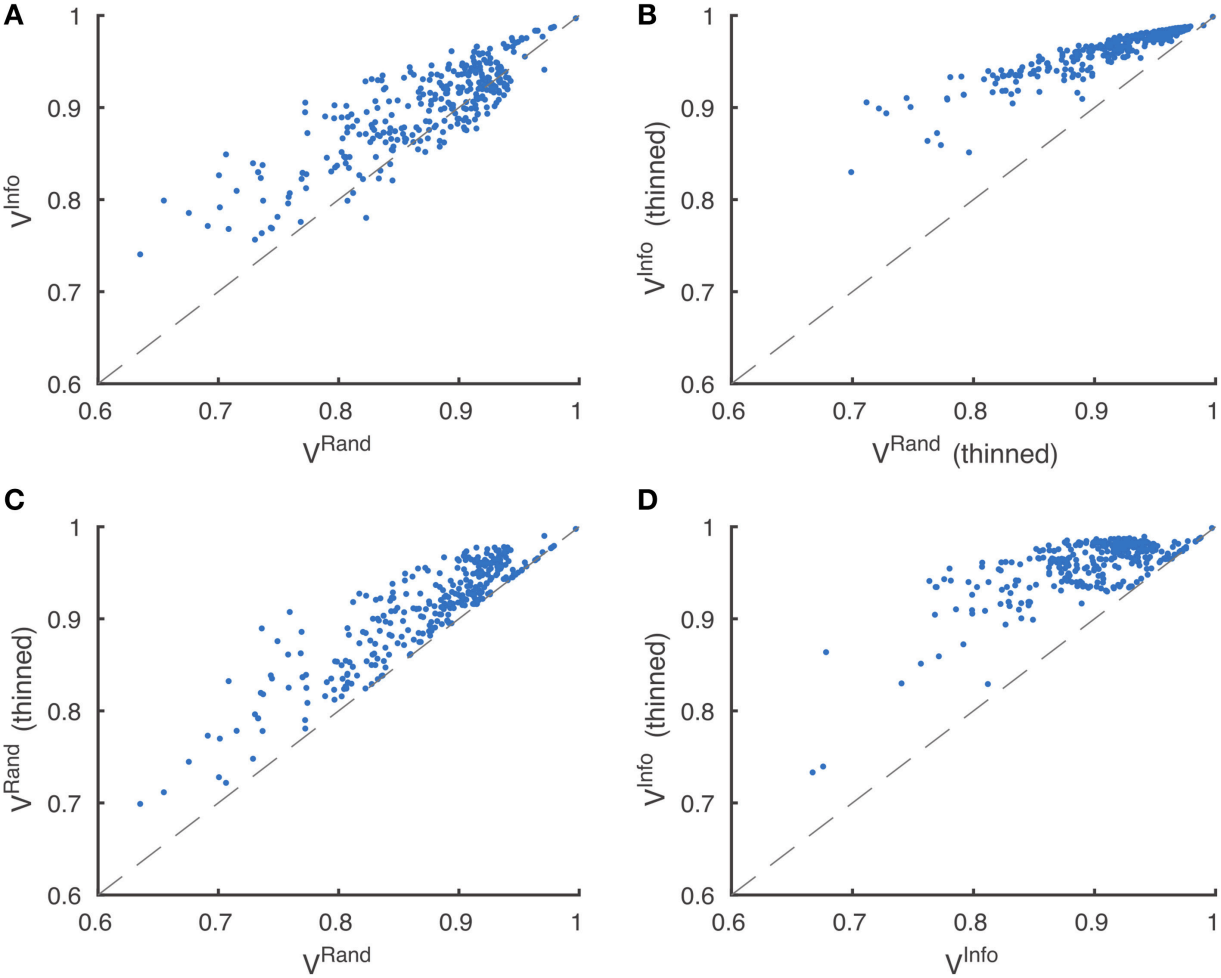
**Metric robustness to thinning**. **(A)** Rand (*V*^Rand^) and information theoretic (*V*^Info^) scoring measures produce similar rankings, Spearman correlation ρ = 0.80. **(B)** This correlation is greatly increased by post-processing boundaries by thinning, Spearman correlation ρ = 0.94. **(C,D)** Thinning of boundaries almost universally improves Rand and information theoretic scoring measures. *V*^Rand^ rankings are more robust to thinning, **(C)** Spearman correlation ρ = 0.89, compared to *V*^Info^ rankings, **(D)** Spearman correlation ρ = 0.59.

Through visual inspection, we found that the boundary maps predicted by BlackEagles and MLL-ETH had markedly wider borders than the boundary maps of other algorithms. We hypothesized that such border variations were the source of the ranking differences. We had already taken one step to improve the robustness of scoring to border variations, which was to compute both Rand and information theoretic scores after foreground-restriction (Section 2.4). We experimented with a further step to improve robustness, which was to thin the borders of all submitted boundary maps in a way that was guaranteed to not merge objects. After this step, the borders in all boundary maps were the same width (about one pixel). Then we computed foreground-restricted scores as before. Inspection of Table 2 shows that the Rand and information theoretic rankings were more similar to each other after border thinning. To quantify this effect, we measured Spearman's rank-order correlation between the different rankings. The rank-order correlation between the Rand and information theoretic rankings increased from 0.80 to 0.94 after border thinning. Graphs of information theoretic vs. Rand scores are provided in Figure 5.

Spearman's rank-order correlation of Rand rankings before thinning and after thinning was 0.89, while the rank-order correlation between the information theoretic rankings before thinning and after thinning was only 0.59. This suggests that the Rand scoring is more robust to border variations than information theoretic scoring. However, our results suggest that neither scoring system is satisfactory without border thinning.

The best submissions after ISBI'12 did not improve over IDSIA by a statistically significant margin, if scores are computed after border thinning. In other words, the cooperative phase of the challenge achieved substantial improvement according to the original challenge scoring system, but this improvement did not reflect a real improvement in the nuisance metric. Instead, the apparent improvement resulted from the scoring system's lack of robustness to border variations.

### 3.5. The challenge has saturated the limits of 2D segmentation

How close have algorithms come to human performance? To address this question, we also scored two human experts relative to the same ground truth used to score the computer algorithms (Table 1 and Figure 3). This suggests that the algorithms still fell short of human performance. Before border thinning, the top submission in Table 1 was superior to H2. We were suspicious of this finding because of a puzzling asymmetry in the scores of the two human experts: H1 scores higher than H2. When we examined the human segmentations, we realized that H2 had thicker borders than H1. Indeed, the H1 and H2 scores are more similar to each other after border thinning (Table 2), and no algorithm is superior to H2. (H2 still scores lower than H1 relative to the ground truth consensus, because of an asymmetry in the procedure that created the consensus from H1 and H2.)

According to Rand scoring after border thinning (Table 2), the top algorithms are slightly inferior to H2. According to information theoretic scoring after border thinning, the top algorithm scores are essentially statistically indistinguishable from H2. These results point to a limitation arising from the size of our test dataset, but also point to the success of the challenge. The algorithms have reached a level of accuracy where it will now take much larger test datasets to distinguish measurable improvements in accuracy.

**Figure 6:**
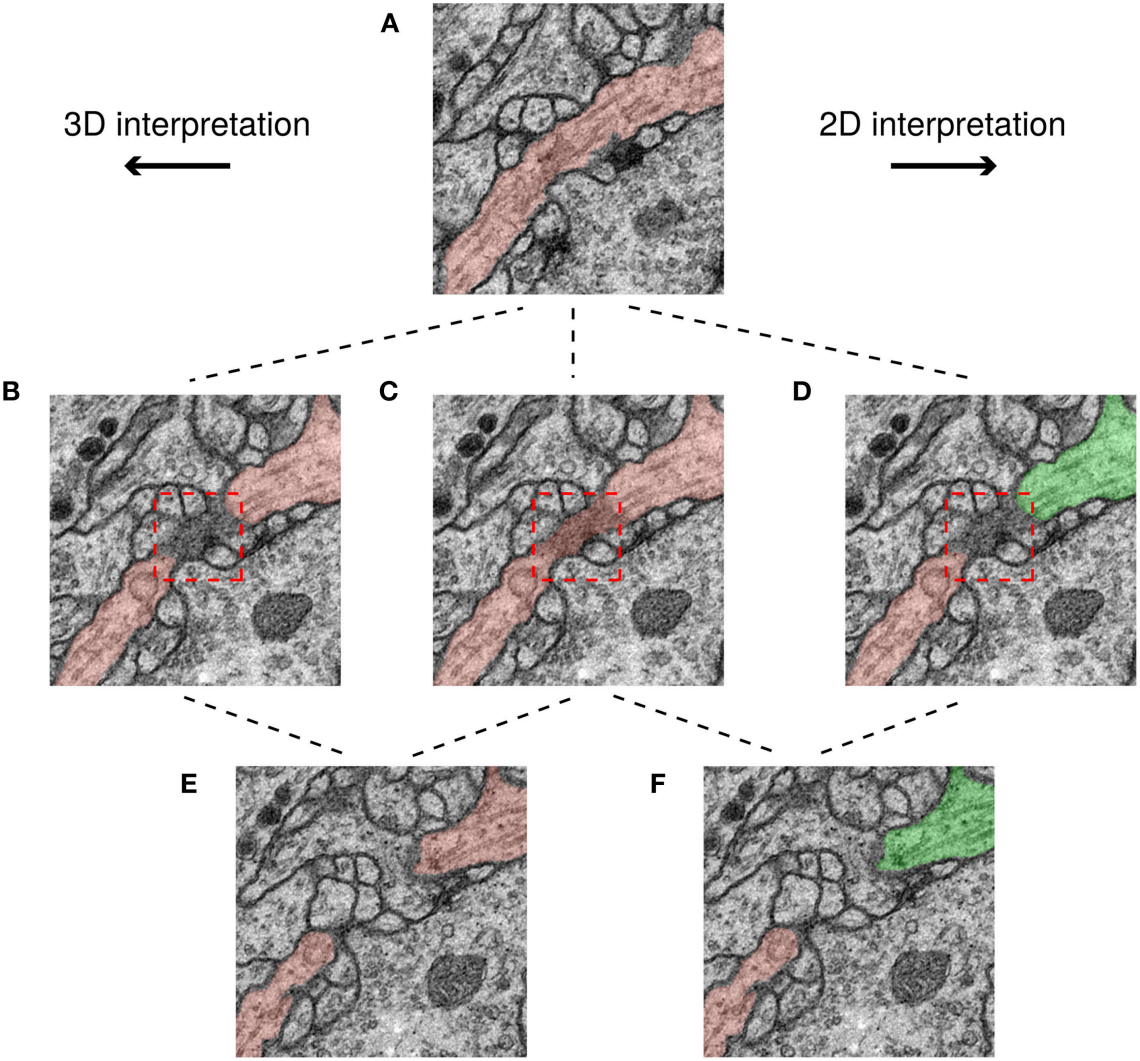
**Minor ambiguities in 3D can become significant in 2D**. Three rows correspond to three successive slices in the image stack and each path shows a possible segmentation of a neuron based on a different interpretation. In the top panel **(A)** the neurite borders are clear and therefore its interpretation is unambiguous. However, in the middle row, a membrane is parallel to the sectioning plane (darkened area in red box), leading to ambiguity. It is unclear whether the darkened area should be a boundary between neurons **(B,D)**, or assigned to a neuron **(C)**. This ambiguity has no topological consequences in 3D unlike in 2D, where the neuron can be assigned to just one segment **(C)**, or two **(D)**. Finally, in the 3D interpretation the two cross sections in **(E)** have the same color because they are connected with each other through previous slices, while in the 2D interpretation, the two cross sections in **(F)** have different colors because they are not connected to each other in this slice.

We examined the differences between H2 and the ground truth consensus, and found that they are mainly due to ambiguities created by scoring the challenge in 2D. An example is shown in Figure 6. The red box shows a region where a cell membrane runs parallel to the sectioning plane and so appears indistinct. The ambiguity of this region does not significantly change the 3D interpretation. However, the ambiguity is severe in 2D, because it affects whether two cross sections should be split or merged.

To summarize, the score of a top algorithm relative to the consensus of two human experts is approaching the score of one human expert relative to the consensus. Human agreement appears limited primarily by ambiguities due to 2D scoring, rather than by genuine ambiguities in the images.

## 4. Discussion

At a 2014 conference on connectomics organized by the Howard Hughes Medical Institute and Max Planck Society, it was obvious that convolutional networks had become a dominant approach for boundary detection in serial EM images. Seven years earlier, the first published reports of this approach (Jain et al., 2007) had been met with skepticism. The turning point in convincing the community may have been the ISBI'12 workshop, when a convolutional network submitted by IDSIA won first place in the challenge described here.

Similarly, convolutional networks were long employed for object recognition (LeCun et al., 1989, 2004), but were resisted by the mainstream computer vision community for decades. Opinions changed with surprising speed after a paper that demonstrated superior performance on the ImageNet challenge (Krizhevsky et al., 2013). Both case studies demonstrate how a challenge with public dataset and scoring system can provide enough objective evidence to persuade a skeptical community to change its opinion dramatically.

Our ISBI'12 challenge also demonstrates the importance of incentivizing both competition and cooperation. Competition dominated the challenge before the winner was declared at the ISBI'12 workshop. Cooperation increased afterwards, chiefly by IDSIA's release of their boundary maps, and resulted in further performance gains. Cooperation by sharing of results was also incentivized in the Netflix challenge. The winner of the yearly progress prize could only collect the prize money after releasing their source code and a description of their algorithm^6^. This insured cooperation during the multi-year competition period.

Our challenge also shows that proper design of the scoring system is crucial for incentivizing real rather than spurious improvements. Retrospectively, we discovered that most of the progress after the ISBI'12 workshop came by exploiting a weakness in our scoring system. We had originally restricted the Rand F-score to the foreground pixels in the ground truth, in order to make the scores more robust to unimportant variations in border width. However, it turned out that our score was still not robust enough. After we applied a border thinning procedure to make all submissions have the same border width, the post-workshop gains mostly vanished.

Nevertheless, our retrospective analysis suggests that the ISBI'12 challenge has succeeded, in the sense that computer-human agreement is approaching human-human agreement, given the limited size of the test dataset. Human experts do not agree perfectly, mainly because of ambiguities induced by 2D scoring of segmentations. The restriction of the challenge to 2D had two rationales. First, we wanted to recruit participants from the entire computer vision community. A 3D challenge might have drawn participants only from the smaller community of medical image analysts. Indeed, our leading submission came from a group (IDSIA) with prior experience mainly in 2D images. Second, many approaches to 3D reconstruction of neurons from serial section EM images rely on 2D segmentation as a first step (Mishchenko et al., 2010; Funke et al., 2012; Kaynig et al., 2015). Therefore, advances in 2D segmentation were expected to yield improvements in 3D reconstruction.

While the ISBI'12 challenge still serves as an accessible introduction to the computational problem, further progress will require a 3D challenge on a larger dataset. We previously attempted to launch one for ISBI'13^7^, but were not successful in attracting many submissions. One possible explanation is that the 2D challenge was easier for participants because they already had experience with 2D images from other domains. We intend to relaunch the 3D challenge with a new dataset, and are working on ways to reduce the barriers to entry. We expect that the general lessons we have learned from the 2D challenge will remain useful.

In closing, it is important to note that the current best 3D reconstruction algorithms still require significant manual proof-reading, itself a crowdsourcing problem, to produce scientifically accurate reconstructions (Helmstaedter et al., 2013; Takemura et al., 2013; Kim et al., 2014). This highlights the fact that our current best error rates of 1–2% are still much too high. Indeed, scientifically accurate fully automated reconstructions require exceedingly high levels of accuracy, with nuisance error rates averaging less than 1 mistake per neuron. However, with recent increases in available training data and computation, and progress in machine learning methods, there is every reason to believe that this goal might be within reach.

## 5. Data sharing

The original training and test image datasets are available in the challenge website. Scripts to run the evaluation metrics are publicly available as part of the Trainable Weka Segmentation library in the open-source imaging platform Fiji (Schindelin et al., 2012).

## Author contributions

IA, JS, AC, and HS are responsible for the organization of the challenge. The training set was labeled by AC. The test set was labeled by DB and IA. The following groups contributed with their algorithms and submissions: DC, AG, LG, and JS (IDSIA); DL, SD, and JB (MLL-ETH); TL, MS, and TT (SCI); LK (CellProfiler); RB and VU (IMMI); XT, CS, and TP (TSC+PP); EB and MU (CLP). The evaluation metrics were designed by IA, HS, and ST, who also wrote the paper. IA coordinated all work.

## Funding

The work of RB and VU was partially supported by project no. CZ.1.07/2.3.00/20.0094. The work of TL, MS, and TT was supported by NIH 1R01NS075314-01 (TT, MHE). HS acknowledges funding from ARO award W911NF-12-1-0594, DARPA award HR0011-14-2-0004, the Human Frontier Science Program, and the Mathers Foundation. ST acknowledges support from the Gatsby Charitable Foundation and the Howard Hughes Medical Institute.

### Conflict of interest statement

The authors declare that the research was conducted in the absence of any commercial or financial relationships that could be construed as a potential conflict of interest.

## Footnotes

^1^http://netflixprize.com/

^2^http://www.heritagehealthprize.com/

^3^https://software.rc.fas.harvard.edu/lichtman/vast/

^4^http://grand-challenge.org/

^5^https://groups.google.com/d/forum/em-segmentation-challenge-isbi-2012

^6^http://www.netflixprize.com/rules

^7^http://brainiac2.mit.edu/SNEMI3D/
