# Supplementary material for: Crowdsourcing the creation of image segmentation algorithms for connectomics
Source: Front Neuroanat. 2015 Nov 5;9:142. doi: 10.3389/fnana.2015.00142 (PMC4633678; doi:10.3389/fnana.2015.00142)
Supplement: Supplementary file 1 [file Presentation1.PDF]

Supplementary Material: Crowdsourcing the creation of image segmentation algorithms for connectomics

Ignacio Arganda-Carreras [1,*], Srinivas C. Turaga[2], Daniel R. Berger[3], Dan Cireşan[4], Alessandro Giusti[4], Luca Maria Gambardella[4], Jürgen Schmidhuber[4], Dmitry Laptev[5], Sarvesh Dwivedi[5], Joachim Buhmann[5], Ting Liu[6], Mojtaba Seyedhosseini[6], Tolga Tasdizen[6], Lee Kamentsky[7], Radim Burget[8], Vaclav Uher[8], Xiao Tan[9], Changming Sun[10], Tuan D. Pham[11], Erhan Bas[2], Mustafa Gokhan Uzunbas[12], Albert Cardona[2], Johannes Schindelin[13], H. Sebastian Seung[14,*]

[1] *Institut Jean-Pierre Bourgin, UMR1318 INRA-AgroParisTech, INRA Centre de Versailles-Grignon, Versailles, France*
[2] *Howard Hughes Medical Institute, Janelia Research Campus, Ashburn, VA, USA*
[3] *Center for Brain Science, Harvard University, Cambridge, MA, USA*
[4] *Swiss AI Lab IDSIA (Dalle Molle Institute for Artificial Intelligence) USI, SUPSI, Lugano, Switzerland*
[5] *ETH Zurich, Zurich, Switzerland*
[6] *Scientific Computing and Imaging Institute, University of Utah, Salt Lake City, UT, USA*
[7] *Imaging Platform, Broad Institute, Cambridge, MA, USA*
[8] *Department of Telecommunications, Faculty of Electrical Engineering and Communication, Brno University of Technology, Brno, Czech Republic*
[9] *University of New South Wales, Canberra, Australia*
[10] *CSIRO Digital Productivity Flagship, North Ryde, NSW, Australia*
[11] *Research Center for Advanced Information Science and Technology, Aizu Research Cluster for Medical Engineering and Informatics, University of Aizu, Aizu-Wakamatsu, Fukushima, Japan*
[12] *Rutgers University, Computer Science Department, NJ, USA*
[13] *Laboratory for Optical and Computational Instrumentation, University of Wisconsin-Madison, Madison, WI, USA*
[14] *Princeton Neuroscience Institute, Princeton University, Princeton, NJ, USA*

Correspondence*:
Ignacio Arganda-Carreras
Institut Jean-Pierre Bourgin, UMR1318 INRA-AgroParisTech, Btiment 2, INRA Centre de Versailles-Grignon, Route de St-Cyr (RD10), 78026 Versailles Cedex, France, Ignacio.Arganda@versailles.inra.fr

H. Sebastian Seung
Princeton Neuroscience Institute, Princeton University, Princeton, NJ 08544, USA, sseung@princeton.edu

1 COMPETITION WEBSITE

The competition website was developed by Nader Shaar using Drupal 7[1]. The website describes the challenge, its motivation, objectives, relevant dates and rules of participation. Without registering in the system, the visitors can access all the challenge information except the image data and the details of the individual submissions. After registration, the participants can download the training and test datasets, submit their own results and navigate through the leading submissions ranking. The only requirement for registration is to fill the on-line registration form with a valid e-mail address and either a new or an existing group name. Different users can belong to the same group. This way, participants from the same lab or institution are grouped together and can submit results from different accounts. Two leaderboards are displayed at any time: one containing the leading groups with their all-time best segmentation results, and one showing the leading submissions. Both boards are sorted in ascending foreground-restricted Rand error. All regular users have access to the information or each individual submission (user, group, date, metric errors and method description) but not to its image data.

The system for scoring submissions is semi-automatic. Each time a new result is uploaded, the system administrator receives an automatic e-mail with the submission details. If the submission passes basic quality control (to screen for corrupted files or unintentional submissions of wrong images), the administrator downloads and scores the submission using a private portion of the test dataset and the three public challenge metrics (pixel error, warping error and foreground-restricted Rand error). The public leaderboards are then automatically updated with the new scores and the user is notified by email.

2 LIMITATIONS OF INITIAL METRICS

During the competition, we quantified the performance of each team by means of three metrics: pixel error, warping error and foreground-restricted Rand score. In the course of the analysis of the results we found strong limitations in the pixel and warping errors that led us to reject them as suitable metrics. Next, we define both rejected metrics and show their flaws with respect to other metrics using extreme cases.

2.1 PIXEL ERROR

Performance at boundary detection can be quantified using the precision-recall (PR) framework (Martin et al., 2004). For a binary classification problem, PR is preferred over the receiver operating characteristic (ROC) framework when there is an imbalance between positive and negative examples. According to convention, positive examples are scarcer than negative ones. In this dataset, boundary pixels are roughly 20% of the image, so they are the positive class, while non-boundary or interior pixels are the negative class. Pixels are divided into four categories, depending on the correspondence between the ground truth and the prediction.

Most entries to the competition submitted a binary-valued boundary map. For these entries, we computed a single precision-recall pair of scores. Some entries submitted a probabilistic boundary map, with values in the unit interval. For these entries, we thresholded the probabilistic boundary map to produce a binary-valued boundary map, and computed precision and recall. Then we varied the threshold to produce a graph of precision versus recall. This PR curve visually displays the tradeoff between precision and recall, or between false positives and false negatives. To summarize an entire PR curve with a single statistic, we use the maximal F-score on the curve.

[1] `https://www.drupal.org/`

2.2 WARPING ERROR

The above quantities are simple to compute, but have a deficiency. A single incorrect boundary pixel may merge two objects into one, or split one object into two. Effects on the pixel precision and recall are minor, even though such topological errors are major. This problem can be solved by using concepts of digital topology (Jain et al., 2010). We can "warp" the ground truth binary labels L^* by flipping a sequence of pixels at *simple points*. A *simple point* is a location on a binary image where the pixel value can be flipped (changed to its complementary value) without changing the topology of the image. We define the "best warping" of L^* onto a predicted labeling L as the warping that minimizes the number of pixel differences between them. An approximation to the best warping can be computed by a greedy sequential procedure. For the best warping of L^* the pixel disagreements are non-simple points, which can be classified into six kinds of topological errors: merges, splits, hole addition and deletion, and object addition and deletion.

Here we penalize only pixels belonging to merge and split errors, which are the most problematic kinds of topological errors when reconstructing neurons. Only these pixels are counted as false positives and false negatives when computing the precision and recall after warping.

2.3 EXTREME CASES

A good test of the robustness of a metric relies on observing its behavior on extreme cases. One extreme case is to predict that all pixels are boundaries (all boundary). Another extreme case is to predict that all pixels are interior (all interior). See Table S1, which provides with interesting and relevant information.

First, predicting all boundary has perfect performance according to the pixel F-score after warping. This spurious result arises because warping the ground truth to match the prediction causes the boundaries in the ground truth to expand. Ultimately, the interior of each object is reduced to a single pixel. The only remaining topological errors are object deletions, which are not penalized at all. (As explained earlier, we use a version of the warping error that includes only splits and mergers.) This problem could be fixed by constraining the warping, for example by a mask of several pixels around the boundaries. This behavior invalidates the warping error as it is for the challenge evaluation purposes, although the metric remains very informative to localize and classify topological errors in segmentations that are close to the ground truth.

Regarding the pixel classification error, the all interior image produces the minimum F-score (0.0) given that the F-score of pixel similarity is calculated based on the true positives and the image has only negative samples. However, the all boundary image is not penalized the same way (given the small proportion of membrane pixels with respect to non-membrane pixels) and its value remains reasonably low.

Finally, the foreground-restricted Rand score strongly penalizes both types of images. As already explained in Section 2.4 (main manuscript), the background pixels of the ground truth are not considered in the error calculation and that is the reason a full foreground input does not produce the minimum possible score. After analyzing these results, the challenge organizers decided to announce the F-score of the foreground-restricted Rand as the final metric based on which the winner of the competition would be determined.

3 COMPARING RAND AND INFORMATION-THEORETIC SCORING MEASURES

Suppose that S is the predicted segmentation and T is the ground truth. Let $S_{\alpha i}$ indicate whether pixel α is assigned to segment i in the predicted segmentation, and let $T_{\beta j}$ indicate whether pixel β is assigned to segment j in the true segmentation. Define p_{ij} as the fraction of pixels assigned to segment i in segmentation S and segment j in segmentation T:

Supplementary Table 1. Test of robustness for each evaluation metric comparing pixel F-score, warping F-score, foreground-restricted Rand score (V^{Rand}) and foreground-restricted information theoretic score (V^{Info}).

Method	Pixel F-score	Warping F-score	V^{Rand}	V^{Info}
human 2 vs human 1	0.8284	0.9993	0.9660	0.9405
all interior	0.0	0.0	0.1442	0.0
all boundary	0.4534	**1.0**	0.0002	0.4317

$$p_{ij} = \frac{1}{N} \sum_{\alpha} S_{\alpha i} T_{\alpha j} = \frac{1}{N} \left(S^T T \right)_{ij}$$

Also define s_i as the fraction of pixels in segmentation S assigned to segment i, and t_j the fraction of pixels in segmentation T assigned to segment j, or

$$s_i = \frac{1}{N} \sum_{\alpha} S_{\alpha i} = \frac{1}{N} \sum_{\alpha} S_{\alpha i}^2 = \frac{1}{N} (S^T S)_{ii}$$

Notice that these are normalized probability distributions, i.e.

$$\sum_i s_i = \sum_j t_j = \sum_{i,j} p_{ij} = 1$$

and satisfying

$$s_i = \sum_j p_{ij} \qquad t_j = \sum_i p_{ij}$$

3.1 RAND-DERIVED SCORING MEASURES

Note that $\left(SS^T\right)_{\alpha\beta}$ indicates whether α and β belong to the same segment in the predicted segmentation, and $\left(TT^T\right)_{\alpha\beta}$ is analogous for the true segmentation. For pixel pair classification we have

$$\begin{aligned} tp &= \operatorname{Tr} SS^T TT^T = N^2 \sum_{ij} p_{ij}^2 \\ fp + tp &= \operatorname{Tr} SS^T = N^2 \sum_i s_i^2 \\ fn + tp &= \operatorname{Tr} TT^T = N^2 \sum_i t_i^2 \end{aligned}$$

Rand precision is the ratio of true positives (tp) to predicted positives ($fp + tp$), or

$$RP = \frac{\sum_{ij} p_{ij}^2}{\sum_i s_i^2}$$

Rand recall is the ratio of true positives (tp) to ground truth positives ($fn + tp$),

$$RR = \frac{\sum_{ij} p_{ij}^2}{\sum_j t_j^2}$$

Rand F-score (referred to as V^{Rand} in the main text) is defined as the harmonic mean of Rand precision and recall, or

$$RF = V^{\text{Rand}} \quad = \quad \frac{2\sum_{ij} p_{ij}^2}{\sum_i s_i^2 + \sum_j t_j^2}$$

These quantities are equal to 1 when A and B match perfectly.

The Rand F-score $RF = V^{\text{Rand}}$ is related to the Rand error by

$$1 - RF(S;T) = \frac{RE(S;T)}{\sum_i s_i^2 + \sum_j t_j^2}$$

where the Rand error is defined by

$$\begin{aligned} RE(S,T) &= N^{-2}(fp + fn) \\ &= \sum_i s_i^2 + \sum_j t_j^2 - 2\sum_{i,j} p_{ij}^2 \end{aligned}$$

The Rand index is given by 1 minus the Rand error. Note that our definitions of the Rand error and Rand index use N^2 normalization, because we allow pixel pairs to include a pixel and itself. Most conventional definitions use $N(N-1)$ normalization because only distinct pairs are allowed.

3.2 INFORMATION-THEORETIC SCORING MEASURES

The fraction of information in S provided by T can be written as:

$$C(S|T) \quad = \quad \frac{\sum_{ij} p_{ij}\log p_{ij} - \sum_i s_i \log s_i - \sum_j t_j \log t_j}{-\sum_i s_i \log s_i}$$

It is sometimes called an "uncertainty coefficient" but "certainty coefficient" would be more appropriate since a larger value means better prediction. Similarly, the fraction of information in T provided by S can be written as

$$C(T|S) \quad = \quad \frac{\sum_{ij} p_{ij}\log p_{ij} - \sum_i s_i \log s_i - \sum_j t_j \log t_j}{-\sum_j t_j \log t_j}$$

Rand precision and recall are analogous to $C(T|S)$ and $C(S|T)$, respectively. The symmetrically normalized mutual information (referred to as V^{Info} in the main text) can be written as

$$C(S;T) = V^{\text{Info}} \quad = \quad 2\frac{\sum_{ij} p_{ij}\log p_{ij} - \sum_i s_i \log s_i - \sum_j t_j \log t_j}{-\sum_i s_i \log s_i - \sum_j t_j \log t_j}$$

All three of the above quantities are equal to 1 when S and T match perfectly.

$C(S;T)$ is closely related to the variation of information,

$$VI(S;T) = H(S) + H(T) - 2I(S;T)$$

which has been used as a measure of segmentation error (Yang et al., 2008; Arbelaez et al., 2009, 2011). The quantities are related by a normalization and sign change,

$$1 - C(S;T) = \frac{VI(S;T)}{H(S) + H(T)}$$

3.3 COMMON MATHEMATICAL FORM FOR RAND AND INFORMATION-THEORETIC SCORING SCHEMES

The Rand and information-theoretic metrics can both be written as

$$1 - V = \frac{\sum_i f(s_i) + \sum_j f(t_j) - 2\sum_{ij} f(p_{ij})}{\left|\sum_i f(s_i) + \sum_j f(t_j)\right|}$$

where $f_{\text{Info}}(x) = -x \log x$ and $f_{\text{Rand}}(x) = x^2$. This common form suggests that the quantities are similar to each other, and should produce similar rankings.

Based on the different behaviors of this function, we expect that the V^{Rand} will be less sensitive to small differences in the sizes of segments across two segmentations. And the V^{Info} should be less sensitive to differences in the sizes of segments within a segmentation.

Suppose that a segment of size $A = \sum_{i=1}^{n} a_i$ in one segmentation is split into pieces of sizes $a_1, \ldots, a_n$ in another segmentation. The amount of splitting can be quantified using the function

$$\Phi\left(a_1, \ldots, a_n\right) = f\left(\sum_{i=1}^{n} a_i\right) - \sum_{i=1}^{n} f\left(a_i\right)$$

which is nonnegative if f is a convex function. It can be shown that the Rand and information theoretic scores are based on the above function with different choices for f.

$$\Phi_R = \sum_{i \neq j} a_i a_j$$

$$\Phi_I = -\sum_i a_i \log a_i + A \log A$$

If a segment of size A is split into pieces of size Ar_i, it follows that

$$\Phi_R = A^2 \sum_{i \neq j} r_i r_j$$

$$\Phi_I = -A \sum_i r_i \log r_i$$

We predict that Rand scores will be more sensitive to small variations of r around 0, because Φ_R has slope $2A^2$ and Φ_I has infinite slope at $r = 0$. Consistent with this, we observed in the main text that V^{Rand} was less sensitive to variations in border thickness than V^{Info}.

4 SEGMENTATION AND BORDER THINNING

We used two strategies for generating segmentations from the submitted gray-scale boundary maps. The first strategy, used throughout the competition, was to binarize the boundary map at many threshold values and then segment the foreground pixels into connected components using 4-connectivity. This generated a hierarchy of nested segmentations, each corresponding to a different threshold.

The second strategy, employed in the retrospective analysis presented here, involved applying a different algorithm to generate the segmentation from the binarized boundary map. Here, we used MATLAB's watershed algorithm, again with 4-connectivity. This approach has the benefit of thinning the boundaries between the segments until the boundaries are only one-pixel wide.

As shown in Section 3.4 and Table 2, almost all scores improved after border thinning, mainly through improvements in split scores. To understand why, note that the opposite change of widening borders creates new border pixels by splitting them off from existing segments. Therefore border thinning has the effect of reducing split errors. Regarding human performance, even after border thinning, H1 remains superior to H2 in Table 2. This is likely because the ground truth T is an edited version of H1, in which some of the topological disagreements with H2 were resolved.

5 PARTICIPANT METHODS

More than a hundred groups and individuals registered for the challenge and downloaded the datasets. Of these, 17 uploaded their results and 7 sent their results and methods to be included in this work. Fig. S1 shows the result of each method in the same area of the test dataset and highlights their topological mistakes with respect to the ground truth. A brief description of each method is presented next.

5.1 METHOD 1: IDSIA

This method approaches the problem of supervised segmentation of neural membranes in EM stacks by means of a Deep Neural Network (DNN) to classify pixels (Ciresan et al., 2012). A DNN, as any convolutional neural network, is a type of artificial neural network where nodes and edges are image valued. Its design emulates the animal visual cortex, where a complex arrangement of cells are sensitive to small sub-regions of the visual field and act as local filters over the input space. The network computes the probability of a pixel being a part of the membrane, using as input the image intensities in a square window centered on the pixel itself. The image is then segmented by classifying all of its pixels, followed by mild post-processing. All available slices of the training stack are used for training the classifier. For each slice, they use all membrane pixels as positive examples (on average, about 50,000), and the same amount of pixels randomly sampled (without repetitions) among all non-membrane pixels. This amounts to 3 million training examples in total, in which both classes are equally represented. Because the appearance of membranes is not affected by their direction, before each training epoch they further augment the training set by randomly mirroring each instance, and/or rotating it by $\pm 90°$. Finally, because each class is equally represented in the training set but not in the testing data, the network outputs tend to severely overestimate the membrane probability. To fix this issue, a polynomial function post-processor is applied to the network outputs. Its coefficients are computed as follows. A separate network is trained on a subset of the training slices and tested on the remaining ones (for which ground truth is available). Resulting outputs (a total of 2.6 million instances) are compared to ground truth, to compute the transformation relating the network output value to the actual probability of being a membrane. The resulting function is well approximated by a monotone cubic polynomial, whose coefficients are computed by least-squares fitting. The same function is then used to calibrate the outputs of all trained networks.

5.2 METHOD 2: MLL-ETH

This algorithm by Laptev et al. (2012) starts by constructing a dense correspondence between neighboring sections. For any given section, the previous and next sections are non-linearly warped into it using scale-invariant feature transform (SIFT) flow (Liu et al., 2011). A total of 626 features is created for every pixel, combining 18 filters at 5 different scales, including Radon-like and ray features, line filter transforms and all the components of the SIFT histogram. The final number of features increases to 1878 by incorporating the features from neighboring sections. The probability of a pixel belonging to a membrane is then evaluated by random forest independently for every pixel. Next, graph cut segmentation is used to take into account the fact that labels of neighboring pixels are more likely to be connected. Finally, as a post-processing procedure two steps are performed iteratively: region removing and line filter transform. Region removing is performed by a series of thresholding operations based on region properties such as area, solidity, Euler Number and eccentricity (thresholding parameters are chosen by hand). The line filter transform makes segmentation results smoother and fills the gaps between membrane segments.

5.3 METHOD 3: SCI

To automatically segment the neural structures this method applies a two-step approach. The authors propose a segmentation framework that exploits a pixel classifier (Seyedhosseini et al., 2011) followed by a watershed merge tree (Liu et al., 2012) for 2D neuron segmentation and membrane detection. First, a series of Multi-Layer Perceptron Artificial Neural Networks (Jurrus et al., 2010) is trained to generate the probability maps of membranes. This pixel classifier takes advantage of multi-scale contextual information and multi-scale Radon-like features (Seyedhosseini et al., 2011) to improve the performance of a conventional series classifier (Jurrus et al., 2010). Though the performance of this pixel classifier is good with regards to the pixel error, the Rand error can be high due to some small gaps between big cells in the EM images. To address this problem, a watershed merge tree (Liu et al., 2012) is built to perform a hierarchical region merging on the watershed transform of the probability maps. A boundary classifier is finally learned with non-local image features to predict each potential merge in the tree, upon which merge decisions are made with consistency constraints to generate the final segmentation. More recently, the authors proposed the Cascaded Hierarchical Model that learns to segment membranes from contextual information in a hierarchical framework (Seyedhosseini et al., 2013). In combination with a modified hierarchical merge tree region merging procedure (Liu et al., 2014), the overall segmentation results are further improved.

5.4 METHOD 4: CELLPROFILER

This method consists of a pipeline of several steps implemented in the open-source tool CellProfiler (Kamentsky et al., 2011). The individual stack planes are first normalized to a uniform intensity by assigning each pixel a value between zero and one based on its rank order. A feature vector is then developed for each pixel in the plane with 1083 features per pixel. The feature vector samples at three different scales: a pixel-level scale designed to detect fine-grained texture in the immediate neighborhood of a pixel, a scale designed to sample the gradient magnitude at roughly the typical membrane width, and a larger scale designed to sample the neighborhood of a mitochondrion or membrane interior. Boundaries were handled by reflecting the image, both when performing Gaussian convolutions and when sampling for the feature vector. A random forest classifier was trained on a subset of the training volume and then the subset was augmented with misclassified pixels and trained for a second round. The classifier was then used to score the test volume, resulting in an initial scoring of each pixel to produce a classification image. The classification image is processed to find seeds for a watershed segmentation (Vincent and Soille, 1991). Finally, the watershed segmentation is post-processed to remove boundaries where there is little evidence for membrane in the classification image and to generate boundaries between catchment basins.

5.5 METHOD 5: IMMI

This method by Uher and Burget (2012) starts by equalizing the histograms of all training and test images in order to reduce the intensity variability between slices. Then, training points are manually selected in order to give higher priority to areas where there is risk of not recognizing the membrane or merging membrane with the mitochondria. Next, different features are extracted from each pixel based on a set of classic filters and used to train a support vector machine (SVM) with dot kernel. In particular, the features include image equalization, several filters (Gaussian blur, edge detection, median, minimum and variance) at different scales and binary skeletonization. A genetic algorithm optimization is used to optimize the parameters of the transforms used. Finally, the probability image provided by the SVM is segmented using the statistical region merging method (Nock and Nielsen, 2004), and from each segment several segment-level features are extracted and labeled as wanted or unwanted by a decision tree. Unwanted objects are then removed from the final result.

5.6 METHOD 6: TSC+PP

This algorithm by Tan et al. (2012) starts by an initial pixel classification which includes adaptive threshold classification using edge strength (ADTES), and dark blob location. Then dark blobs which may be incorrectly classified into the membrane class are revisited and eliminated by checking its co-occurrence in the neighboring images of the stack. After that, a set of features, including filters at different scales, the attribute opening filter, and the distance transform of the result obtained from the ADTES, are extracted and used to train a SVM for further pixel classification. Since the classes are imbalanced, 25,000 samples of each class are randomly selected among all input pixels to train the SVM. Finally, post-processing is applied on the probability map obtained from the SVM to recover some membrane structures which may have been misclassified as non-membrane and to eliminate some dark regions which are incorrectly classified as membrane.

5.7 METHOD 7: CLP

In order to extract useful information from EM images, this method by Bas and Uzunbas (2012) first characterizes regions structurally as well as contextually. The approach consists of a multistage decision mechanism that utilizes underlying differential geometric properties of objects in a biologically inherited framework. A feature selection procedure to select most relevant features characterizes distinct regions, such as membrane, cytoplasm and outliers. Similar to a topographic map, a random forest classifier highlights mountain ridge-like structures, e.g., membranes, as well as plateaus, e.g., cytoplasm. Principal surface analysis is applied to extract the underlying geometry of structures on this topographic map, particularly membrane-like structures. This unsupervised technique returns highly sparse yet accurate low dimensional representation of the data, particularly for characterizing membrane-like regions. A task-specific, second stage decision mechanism is employed to distinguish contextually different mitochondria and cell boundary membranes. This second stage learning/decision mechanism is based on the appearance, the initial topographic map with its low dimensional reconstruction and expert supervision on different types of membranes.

REFERENCES

Martin D, Fowlkes C, Malik J. Learning to detect natural image boundaries using local brightness, color, and texture cues. *Pattern Analysis and Machine Intelligence, IEEE Transactions on* **26** (2004) 530 –549. doi:10.1109/TPAMI.2004.1273918.

Jain V, Bollmann B, Richardson M, Berger DR, Helmstaedter MN, Briggman KL, et al. Boundary learning by optimization with topological constraints. *2010 IEEE Conference On Computer Vision and Pattern Recognition (CVPR)* (IEEE Comp Soc) (2010), 2488–2495. doi:{10.1109/CVPR.2010.5539950}.

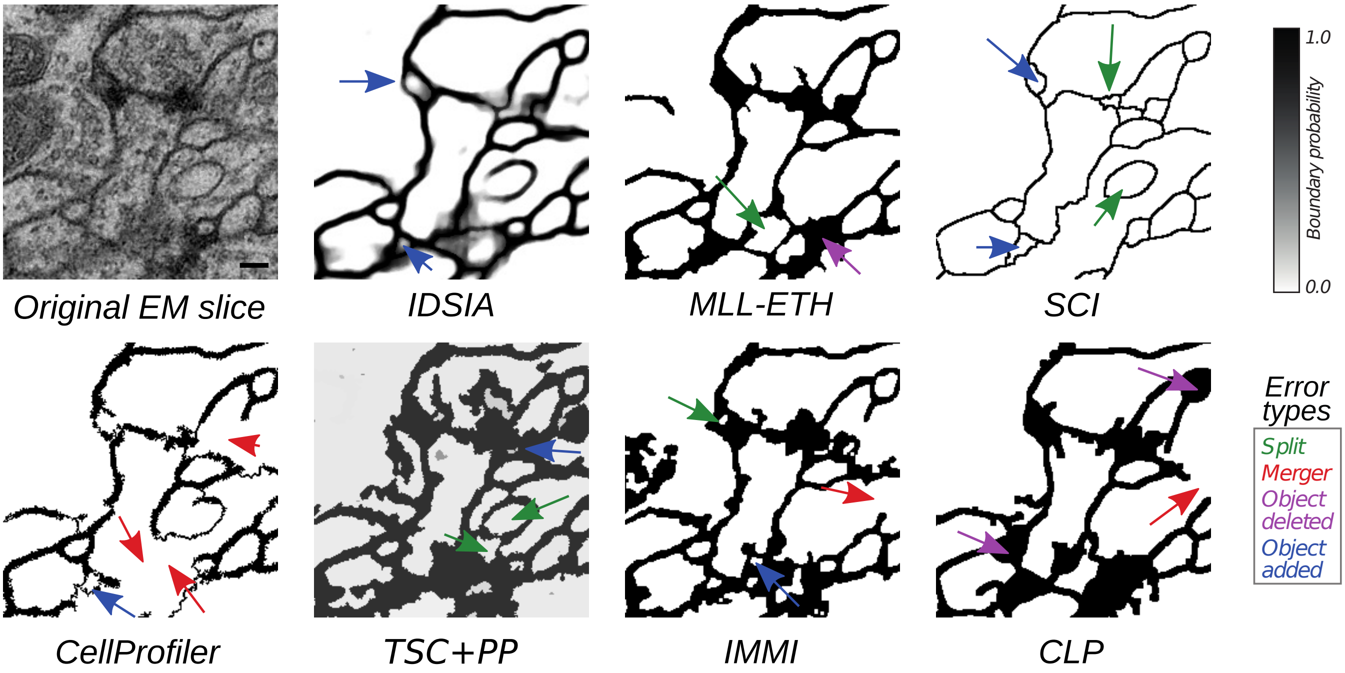

Supplementary Figure 1. Probability maps of the participant methods on a zoomed area of a test slice. After the original test image, the probability maps of each method are sorted from left to right and from top to bottom based on the final ranking of the challenge. Segmentation errors are marked by arrows colored based on the type of mistake: split (green), merge (red), omission (magenta) and addition (blue). Scale bar = 100 nm.

Yang AY, Wright J, Ma Y, Sastry SS. Unsupervised segmentation of natural images via lossy data compression. *Computer Vision and Image Understanding* **110** (2008) 212–225.

Arbelaez P, Maire M, Fowlkes C, Malik J. From contours to regions: An empirical evaluation. *Computer Vision and Pattern Recognition (CVPR), 2009 IEEE Conference on* (2009), 2294 –2301. doi:10.1109/CVPR.2009.5206707.

Arbelaez P, Maire M, Fowlkes C, Malik J. Contour detection and hierarchical image segmentation. *Pattern Analysis and Machine Intelligence, IEEE Transactions on* **33** (2011) 898–916. doi:10.1109/TPAMI.2010.161.

Ciresan DC, Meier U, Schmidhuber J. Multi-column deep neural networks for image classification. *Computer Vision and Pattern Recognition* (2012), 3642–3649.

Laptev D, Vezhnevets A, Dwivedi S, Buhmann JM. Anisotropic ssTEM image segmentation using dense correspondence across sections. *Proceedings of the 15th International Conference on Medical Image Computing and Computer-Assisted Intervention - Volume Part I* (Berlin, Heidelberg: Springer-Verlag) (2012), MICCAI'12, 323–330. doi:10.1007/978-3-642-33415-3_40.

Liu C, Yuen J, Torralba A. SIFT flow: Dense correspondence across scenes and its applications. *Pattern Analysis and Machine Intelligence, IEEE Transactions on* **33** (2011) 978 –994. doi:10.1109/TPAMI.2010.147.

Seyedhosseini M, Kumar R, Jurrus E, Giuly R, Ellisman M, Pfister H, et al. Detection of neuron membranes in electron microscopy images using multi-scale context and Radon-like features. *Proceedings of the 14th International Conference on Medical Image Computing and Computer-Assisted Intervention - Volume Part I* (Berlin, Heidelberg: Springer-Verlag) (2011), MICCAI'11, 670–677. doi:10.1007/978-3-642-23623-5_84.

Liu T, Jurrus E, Seyedhosseini M, Ellisman M, Tasdizen T. Watershed merge tree classification for electron microscopy image segmentation. *Proceedings of the 21st International Conference on Pattern Recognition (ICPR)* (2012), 133–137.

Jurrus E, Paiva AR, Watanabe S, Anderson JR, Jones BW, Whitaker RT, et al. Detection of neuron membranes in electron microscopy images using a serial neural network architecture. *Medical Image Analysis* **14** (2010) 770–783. doi:10.1016/j.media.2010.06.002.

Seyedhosseini M, Sajjadi M, Tasdizen T. Image segmentation with cascaded hierarchical models and logistic disjunctive normal networks. *Computer Vision (ICCV), 2013 IEEE International Conference on* (IEEE) (2013), 2168–2175. doi:10.1109/ICCV.2013.269.

Liu T, Jones C, Seyedhosseini M, Tasdizen T. A modular hierarchical approach to 3D electron microscopy image segmentation. *Journal of neuroscience methods* **226** (2014) 88–102. doi:10.1016/j.jneumeth.2014.01.022.

Kamentsky L, Jones TR, Fraser A, Bray MA, Logan DJ, Madden KL, et al. Improved structure, function and compatibility for CellProfiler: modular high-throughput image analysis software. *Bioinformatics* **27** (2011) 1179–1180. doi:10.1093/bioinformatics/btr095.

Vincent L, Soille P. Watersheds in digital spaces: An efficient algorithm based on immersion simulations. *IEEE Trans. Pattern Anal. Mach. Intell.* **13** (1991) 583–598. doi:10.1109/34.87344.

Uher V, Burget R. Automatic 3D segmentation of human brain images using data-mining techniques. *35th International Conference on Telecommunications and Signal Processing* (IEEE) (2012), 578–580. doi:10.1109/TSP.2012.6256362.

Nock R, Nielsen F. Statistical region merging. *Pattern Analysis and Machine Intelligence, IEEE Transactions on* **26** (2004) 1452 –1458. doi:10.1109/TPAMI.2004.110.

Tan X, Sun C, Pham TD. Membrane extraction using two-step classifier and post-processing (2012).

Bas E, Uzunbas MG. Contextual grouping in a concept: a multistage decision strategy for EM segmentation (2012).
